# Postextrastimulus Termination of Ventricular Tachycardia: An Indicator of a Good Ablation Site?

**DOI:** 10.19102/icrm.2026.17072

**Published:** 2026-07-15

**Authors:** Meryem Kara, Ozcan Ozeke, Elif Hande Ozcan Cetin, Duygu Kocyigit Burunkaya, Muhammet Genes, Ahmet Korkmaz, Firat Ozcan, Serkan Cay, Dursun Aras, Serkan Topaloglu

**Affiliations:** 1 Department of Cardiology, University of Health Sciences, Ankara Bilkent City Hospital, Ankara, Turkey; 2 Department of Cardiology, Mehmet Akif İnan Training and Research Hospital, Sanliurfa, Turkey; 3 Department of Cardiology, İstanbul Medipol University, İstanbul, Turkey

**Keywords:** Nonglobal capture, nonglobal capture, tachycardia termination without global propagation, ventricular tachycardia

## Abstract

Entrainment mapping, or continuous resetting of a re-entrant tachycardia, provides a powerful tool for distinguishing between focal and re-entrant ventricular tachycardias (VTs) and for identifying critical circuit components, such as the entrance, critical isthmus (CI), and exit sites. However, this technique is not always feasible and, in some cases, may itself terminate VT during the entrainment attempt. Whereas local capture of the CI without global ventricular capture (nonglobal capture [NGC]) can terminate VT by creating a bidirectional block (orthodromic or nonorthodromic capture) within the CI, the VT termination may also occur due to a rate-dependent block in the slow-conduction zone during pacing or as a result of spontaneous or mechanical trauma–related ventricular extrasystoles. Such NGC-related termination does not carry the same mechanistic implications as termination due to NGC. Although this termination is usually observed incidentally, it can also be demonstrated intentionally, eg, by delivering a single extrastimulus during tachycardia, as shown in the present case. Therefore, a careful analysis of the response to entrainment is essential.

## Case presentation

A 61-year-old man with a history of ischemic cardiomyopathy (left ventricular ejection fraction, 20%) was referred for ablation of recurrent ventricular tachycardia (VT). Eight years earlier, he had experienced a myocardial infarction and undergone coronary bypass surgery. Electroanatomic mapping of the endocardium revealed an extensive dense scar extending from the base toward the apex along the anterior and septal walls. Within the apical portion of the scar, there was a localized region with late potentials and split electrograms (EGMs). The clinical VT was spontaneously induced **(Figure 1, Video 1)** and was hemodynamically tolerated. The VT origin was estimated to be in the inferoseptal mid-left ventricle, given the QRS morphology of a right bundle branch block with a superior axis, with a cycle length of 440 ms at the blue point **(Video 1)**. In addition to the spontaneously occurring episodes **(Figure 1)**, single ventricular extrastimuli were delivered from this site to determine whether VT termination would occur **(Figure 2, Video 2)**. Based on this response, we questioned whether the pacing site was in or out of the re-entrant circuit.

**Figure 1:**
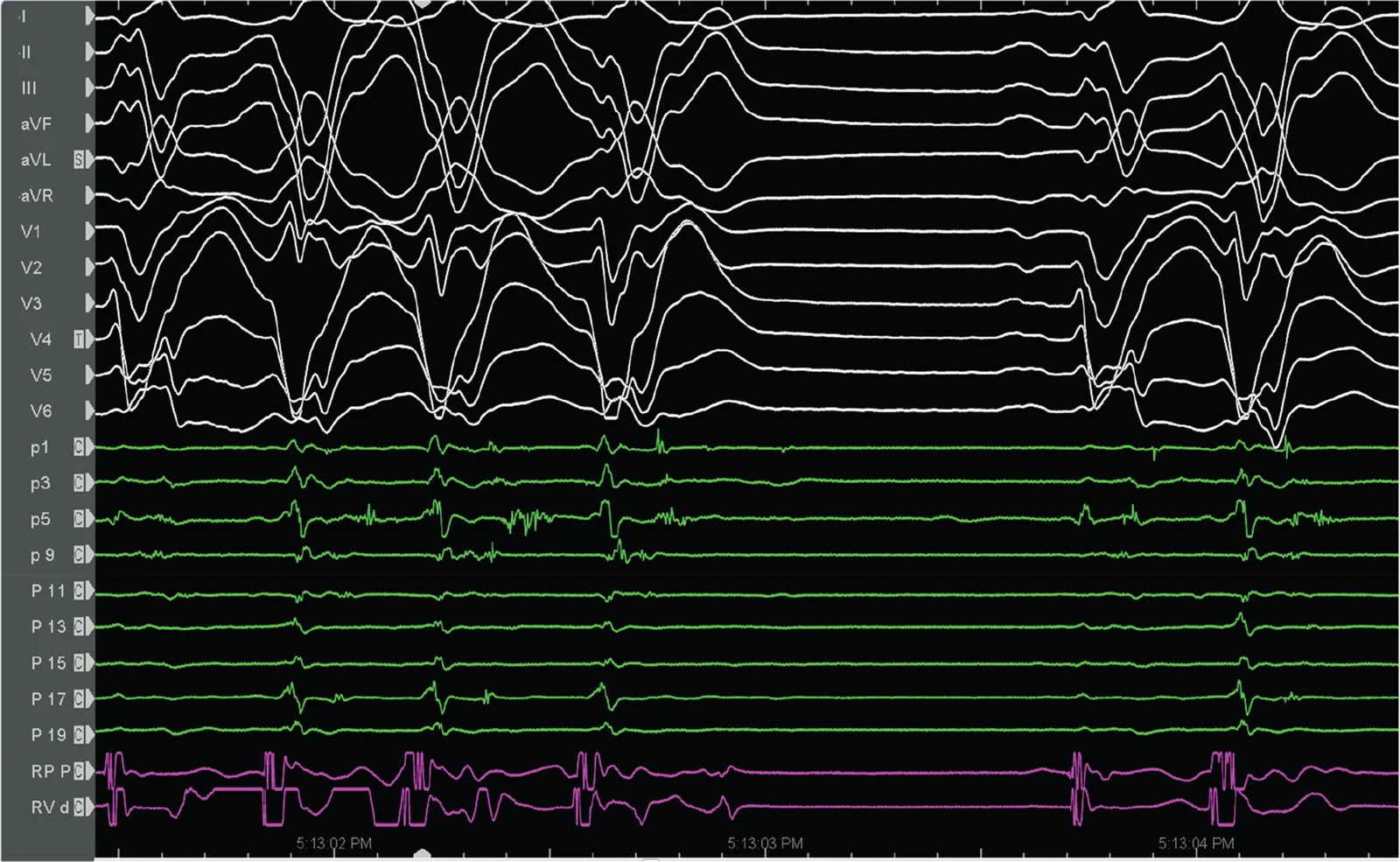
Termination response to single spontaneous ventricular extrasystole during ventricular tachycardia.

**Figure 2:**
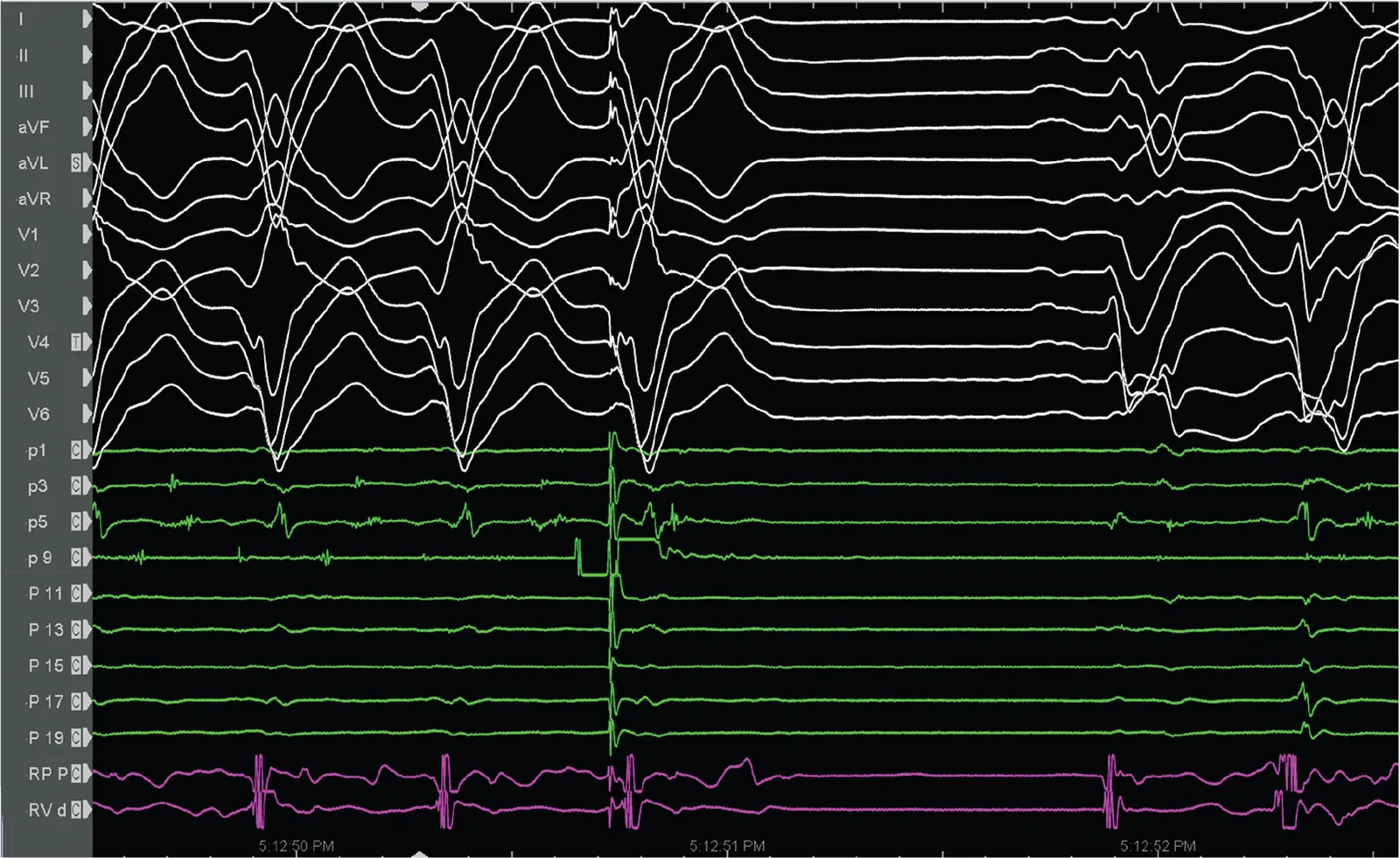
Termination response to a single intentionally given ventricular extrasystole during ventricular tachycardia.

## Discussion

In the setting of structural heart disease, VT is typically sustained by a re-entrant mechanism.^1–3^ Successful VT catheter ablation depends on identifying the critical tissues that sustain the arrhythmia. Four mapping techniques consistently play a role in successful VT ablation: activation mapping, entrainment mapping, pace mapping, and substrate mapping. Entrainment mapping, or continuous resetting of a re-entrant tachycardia, provides a powerful tool for distinguishing between focal and re-entrant VT, as well as identifying critical circuit components such as the entrance, critical isthmus (CI), and exit sites. The detection of the ideal site for ablation within a central CI is a mid-diastolic potential during VT that exhibits concealed fusion with a post-pacing interval equal to the tachycardia cycle length. Thus, entrainment mapping helps to identify the CI, but it is not always feasible and, in some cases, terminates VT.^4^ Indeed, local capture of the CI without global ventricular capture (nonglobal capture [NGC]) can terminate VT by resulting in a bidirectional block (orthodromic or nonorthodromic capture) within the CI.^5–8^ However, termination might also occur due to a rate-dependent block in the slow-conduction zone during pacing. Furthermore, all these pacing attempts rely on consistent capture of tissue with each stimulus. Pacing output is set to minimal output permitting consistent capture. If an arrhythmia terminates during overdrive pacing but several stimuli that should capture do not, then the termination might have been spontaneous or been caused by mechanical trauma **(Figure 3)**; such events do not carry the same implications as termination by NGC. Although this termination is usually observed incidentally, it can also be demonstrated intentionally, eg, by delivering a single extrastimulus during tachycardia, as shown in the present case. Indeed, the resetting and entrainment terms are often incorrectly used interchangeably.^9^ While resetting and entrainment have certain things in common, they differ significantly in the ability to characterize the properties of VT. Only resetting, which is the interaction of a single extrastimulus with the tachycardia, can characterize the properties of the VT itself. Entrainment assesses the effect of overdrive pacing on a reset circuit, not the VT itself.^9^

**Figure 3:**
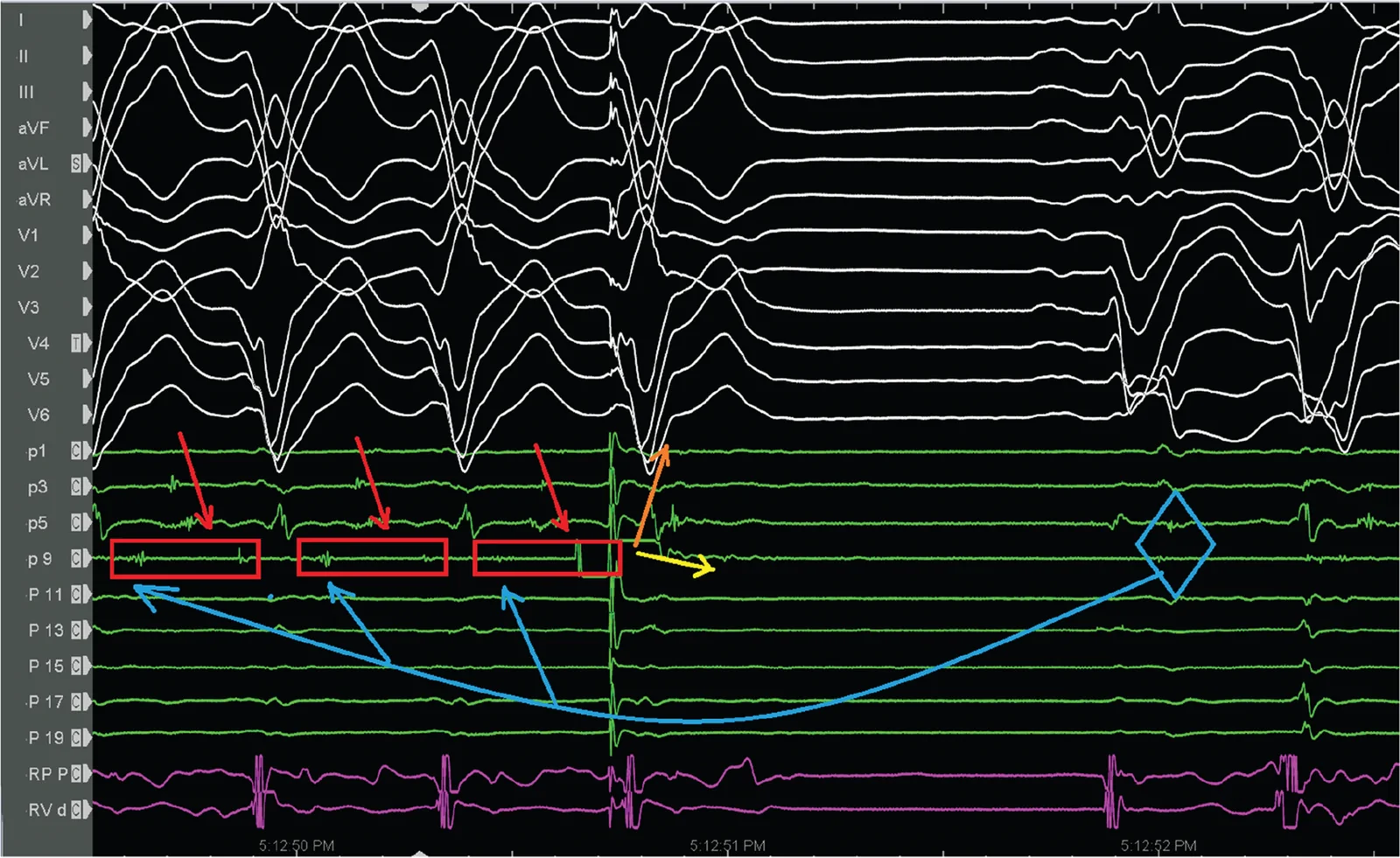
Termination of ventricular tachycardia by a single intentionally delivered ventricular extrastimulus, with local electrogram capture and bidirectional activation (orthodromic, yellow arrow; nonorthodromic, orange arrow). Double potentials recorded from the same electrode (red rectangles in P9) suggest a three-dimensional structure of the V-shaped re-entry circuit.

While “capture” refers to whether the pacing stimulus depolarizes the local myocardium, “global capture” means that the entire QRS complex (ie, the full ventricular myocardium) is activated by the pacing stimulus and the morphology differs from the VT QRS.^10^ On the other hand, NGC means that only a part of the VT circuit or surrounding myocardium is depolarized without altering the overall QRS morphology during VT, supporting CI involvement. VT termination by pacing with NGC is a specific criterion for identifying a critical component of the re-entrant circuit, regardless of whether concealed entrainment can be demonstrated at that site.^5^ It is usually observed almost by chance, but it can also be intentionally demonstrated by introducing a single extrastimulus during tachycardia **(Video 3)**. The entrainment attempt requires the ability to capture an EGM component of interest during overdrive pacing.

In the current tracing, VT was terminated with NGC by a single extrastimulus at this site; however, concealed entrainment could not be assessed due to the termination of the VT. In fact, following delivery of the stimulus (P5 in **Figure 2**), a double potential is observed (P5 in **Figure 2**), suggesting bidirectional conduction block or at least bidirectional local activation **(Figure 3)**, together with apparent orthodromic and antidromic capture.

Of note are the double potentials seen with the QRS complexes after termination of the tachycardia for a sinus speed and a premature ventricular beat (blue square in **Figure 3**). These observations are clinically and electrophysiologically significant, particularly regarding the behavior of late potentials during VT and their alterations in response to stimulation. In general, amplifier saturation from pacing typically obscures the local EGM on the pacing electrodes at the time of pacing; therefore, the careful examination of “adjacent electrode” recordings (P1–3–5 in **Figure 1**) is important to confirm evidence of local capture. Presumably, the stimulus delivered from the EGM channel demonstrating a V-shaped diastolic activation pattern (red rectangles in P9; **Figure 3**) induced a bidirectional block at the exit site of the VT circuit.^11^ When two distinct EGM components are recorded within the same mapping site (highlighted by the red rectangles), this finding is generally interpreted in favor of a line of block. The so-called “V-shaped” activation pattern reflects wavefront propagation around a zone of conduction block rather than through it.^11^ This interpretation is supported by the observation of EGMs that occurred earlier than anticipated and appeared in a reordered sequence, deviating from the expected conduction timing (note the P5 EGM changes in sequence and timing by extrastimulus in **Figures 2 and 3**). Such findings are consistent with bidirectional (orthodromic, yellow arrow; nonorthodromic, orange arrow in **Figure 3**) capture within the CI, suggesting that this site represents a functionally significant component of the re-entry pathway and therefore an optimal target for ablation **(Video 4)**.^5^ Taken together, these observations support the role of this site in sustaining the VT, and ablation rendered the clinical VT non-inducible.

## Supporting information

Supplementary Video 1: Activation mapping shows the re-entry circuit at the inferior left ventricle

Supplementary Video 2: Termination of ventricular tachycardia by a single extrasystole

Supplementary Video 3: Termination of ventricular tachycardia by radiofrequency ablation with gradual prolongation of cyle length

Supplementary Video 4: A serial a single extrasystoles applied during ventricular tachycardia.
